# Enhancement of Natural Killer Cell Cytotoxicity by Sodium/Iodide Symporter Gene-Mediated Radioiodine Pretreatment in Breast Cancer Cells

**DOI:** 10.1371/journal.pone.0070194

**Published:** 2013-08-05

**Authors:** Hae Won Kim, Jung Eun Kim, Mi-Hye Hwang, Yong Hyun Jeon, Sang-Woo Lee, Jaetae Lee, Seok Kil Zeon, Byeong-Cheol Ahn

**Affiliations:** 1 Department of Nuclear Medicine, Kyungpook National University School of Medicine, Daegu, Republic of Korea; 2 Department of Nuclear Medicine, Keimyung University School of Medicine, Daegu, Republic of Korea; Rutgers - New Jersey Medical School, United States of America

## Abstract

A phase II study of NK cell therapy in treatment of patients with recurrent breast cancer has recently been reported. However, because of the complexities of tumor microenvironments, effective therapeutic effects have not been achieved in NK cell therapy. Radioiodine (I-131) therapy inhibits cancer growth by inducing the apoptosis and necrosis of cancer cells. Furthermore, it can modify cancer cell phenotypes and enhance the effect of immunotherapy against cancer cells. The present study showed that I-131 therapy can modulate microenvironment of breast cancer and improve the therapeutic effect by enhancing NK cell cytotoxicity to the tumor cells. The susceptibility of breast cancer cells to NK cell was increased by precedent I-131 treatment *in vitro*. Tumor burden in mice treated with I-131 plus NK cell was significantly lower than that in mice treated with NK cell or I-131 alone. The up-regulation of Fas, DR5 and MIC A/B on irradiated tumor cells could be the explanation for the enhancement of NK cell cytotoxicity to tumor cells. It can be applied to breast cancer patients with iodine avid metastatic lesions that are non-responsive to conventional treatments.

## Introduction

Breast cancer is the most common cancer and the second most common cause of cancer-related death in women, with more than one million cases and nearly 600,000 deaths occurring annually worldwide [1]. Breast cancer is characterized by a distinct pattern of metastasis involving regional lymph nodes, bone, lung, and liver, and the distant metastasis is closely associated with poor prognosis [2]. Once breast cancer has metastasized, it is usually not cured by current therapies, including high dose chemotherapy, likely due to subpopulations of slow-dividing chemoresistant cells present in metastatic cells [3]. In addition, the triple negative breast cancers lack a therapeutic target and have a poor prognosis [4]. Therefore, establishment of new therapeutic strategies is crucial to improving the prognosis of advanced breast cancer.

Tumor-specific immunotherapy offers considerable potential in management of patients with breast cancer; one of the effective immunotherapies is use of natural killer (NK) cells [5]. NK cells, a subset of lymphocytes capable of mediating cytotoxicity against tumor cells and virally infected cells, constitute a key component of the innate immune system [6]. NK cells have been shown to play an important role in controlling the growth of various tumor cell lines in mice [6], [7]. A phase II study of NK cell therapy in treatment of patients with recurrent breast cancer has recently been reported [8]. However, because of the complexities of tumor microenvironments, effective therapeutic effects have not been achieved in NK cell therapy [9], [10].

Radioiodine (I-131) therapy inhibits cancer growth through induction of apoptosis and necrosis of cancer cells [11]. In addition, it was shown that I-131 therapy can modify cancer cell phenotypes and enhance the effect of immunotherapy against cancer cells [12]–[14]. In particular, irradiated cancer cells show up-regulated levels of Fas and tumor necrosis factor–related apoptosis inducing ligand (TRAIL) receptor [15]–[18]. In breast cancer, I-131 therapy can be used, as the majority of breast cancer (70–80%) expresses human sodium/iodide symporter (hNIS), which is a specialized active iodide transporter [11], [18], [19]. hNIS expression and I-131 uptake by breast cancer cells has been suggested to provide supportive evidence for use of I-131 as an additional modality for treatment of breast cancer [20]. Therefore, it is expected that pretreatment with I-131 will result in modification of cancer cell phenotypes and enhance the susceptibility of breast cancer cells to NK cell cytotoxicity.

In this study, we attempted to determine whether Fas and TRAIL receptors of breast cancer cells are up-regulated by I-131 therapy and whether I-131 therapy can enhance NK cell cytotoxicity *in vitro* and *in vivo.*

## Materials and Methods

### Cell Lines

The NK92-MI human NK cell line (NK92-MI) was obtained from the American Type Culture Collection (Rockville, MD, USA). NK92-MI cells were incubated in alpha modification of Eagle’s minimum essential medium (α-MEM; Invitrogen, Carlsbad, CA, USA) supplemented with 2 mM L-glutamine, 0.2 mM inositol, 0.02 mM folic acid, 0.01 mM 2-mercaptoethanol, 10% fetal bovine serum (FBS; Gemini Bio-Products, West Sacramento, CA, USA), and 10% horse serum (Sigma-Aldrich Corporation, St Louis, MO, USA). Human breast cancer cell line MDA-MB 231 (MDA-231) was purchased from the American Type Culture Collection. The MDA-MB 231 cell line coexpressing hNIS and enhanced firefly luciferase (effluc) genes (MDA-231/NF) was kindly provided by J.K Chung (Seoul National University, Seoul, Republic of Korea). MDA-231/NF cells were maintained in Roswell Park Memorial Institute medium (RPMI-1640; Sigma-Aldrich Corporation) supplemented with 10% FBS and 100 U/mL penicillin/streptomycin.

### Animals

Specific pathogen-free six-week-old female BALB/c nude mice (Hamamatsu, Shizuoka, Japan) were used in *in vivo* study. All animal experiment protocols were conducted in accordance with National Institutes of Health guidelines for the care and use of laboratory animals and approved by the Committee for the Handling and Use of Animals of Kyungpook National University.

### RT-PCR Analysis for hNIS and Effluc Genes

MDA-231 and MDA-231/NF cells and homogenized human thyroid tissue were lysed using a Trizol solution (Invitrogen), and total RNA was extracted according to the manufacturer’s instructions. Reverse transcription was performed using a RevertAid First Strand cDNA Synthesis kit (Fermentas, Ontario, Canada). After denaturation of the samples for 1 min at 94°C, 30 cycles for 25s at 94°C, 30 s at 57°C, and 30 s at 72°C were followed with an additional 10 min at 72°C. Two units of Taq DNA polymerase (Takara, Shiga, Japan) using a GeneAmp PCR system (Bio-Rad, Hercules, CA, USA) and the following primers were used: hNIS gene, forward: 5′-CTACGAGTACCTGGAGATGC-3′, reverse: 5′-GTCGCAGTCAGTGTAGAACA-3′; GAPDH, forward: 5′-GCCAAAAGGGTCATCATCTC-3′, reverse: 5′-GATGAGGCAGGGATGATGTT-3′; effluc gene, forward: 5′-GCACAAGGCCATGAAGAGAT-3′, reverse: 5′-CTTCTTGCTCACGAACACCA-3′. Samples were separated by electrophoresis in an ethidium bromide-stained agarose gel.

### I-125 Uptake Assay

The day before iodine uptake, 1×10^5^, 2×10^5^, 4×10^5^, and 8×10^5^ MDA-231 and MDA-231/NF cells were plated in 24-well plates. The level of I-125 uptake was determined by incubation of the cells with 500 µl of Hank’s balanced salt solution (HBSS) containing 0.5% bovine serum albumin (bHBSS), 3.7 kBq carrier-free I-125, and 10 µmol/L sodium iodide (specific activity of 740 MBq/mmol) at 37°C for 30 min. The blocking control study for hNIS was performed in an identical manner, with the exception of the addition of 1mM KClO_4_ to the incubation buffer. After incubation, the cells were washed twice as quickly as possible with ice-cold bHBSS buffer and detached using 500 µL of trypsin. Radioactivity was measured using a gamma-counter (CobraII, Packard, Perkin Elmber, Waltham, MA, USA).

To evaluate the functional expression of the hNIS gene *in vivo*, MDA-231/NF cells (5×10^5^) in phosphate buffered saline (PBS) were implanted subcutaneously into the right flank of three mice. Fourteen days after tumor implantation, Tc-99m pertechnetate SPECT/CT scan was performed using the Inveon small animal imaging system (Siemens Medical Solutions, Knoxville, TN, USA). The mice were placed in a spread-prone position at 40 min after injection of Tc-99m pertechnetate (7.4 MBq/0.2 mL of 0.9% NaCl) into the tail vein, and scanned for 20 min. A 20% window was centered at the 140 keV photopeak of Tc-99m. The 3-D ordered subset expectation–maximization (OSEM) algorithm was used in reconstruction. The voxel size of the image matrix was 0.5×0.5×0.5 mm. All reconstructed images were normalized using a correction matrix derived from a uniform cylindrical phantom image prior to reconstruction.

### Luciferase Assay

In examination of the luciferase assay *in vitro*, 1×10^5^, 2×10^5^, 4×10^5^, and 8×10^5^ MDA-231 and MDA-231/NF cells were plated in 96-well white plates and cultured with Dulbecco’s modified eagle medium (DMEM; Sigma-Aldrich Corporation, St Louis, MO, USA) containing 10% FBS. After 24 hr incubation, 6 µL of D-luciferin (30 mg/mL) was added to each well, followed by measurement of bioluminescence using a microplate luminometer (Molecular Devices, Sunnyvale, CA, USA).

To evaluate the functional expression of the effluc gene *in vivo*, MDA-231/NF cells in PBS were implanted subcutaneously into the right hind-flank (1×10^5^), left hind-flank (3×10^5^), and right fore-flank (9×10^5^) of three mice. After tumor implantation, bioluminescence imaging was performed using the IVIS lumina II imaging system (Caliper, Alameda, CA, USA), which included a highly sensitive CCD camera mounted on a light-tight specimen chamber.

### Phenotype Marker Analysis

To determine the levels of Fas, TRAIL receptor2 (DR5), major histocompatibility complex (MHC) class I chain-related molecule A and B (MIC A/B), and human leukocyte antigen -A, B, and C (HLA-ABC) expression in cancer cells, MDA-231/NF cells were grown in 75 cm^2^ flasks and incubated for 7 hr at 37°C in HBSS only or in HBSS containing 14.8 MBq/5 mL of I-131. The reaction was terminated by removal of the medium containing the radioisotope and by washing cells twice with HBSS. Cells were then grown for three days. These cells were then stained with phycoerythrin (PE)-anti-Fas (BD Biosciences, San Jose, CA, USA), PE-anti-DR5 (eBioscience, San Diego, CA, USA), PE-anti-MIC A/B (BD Biosciences), and PE-anti-HLA-ABC (BD Biosciences). Flow cytometric analysis was performed using a Becton Dickinson FACScan unit using CELL Quest software (Becton Dickinson Immunocytometry Systems, San Jose, CA, USA).

### Cytotoxicity Assay

Cytotoxic activity of NK92-MI cells was assessed using the Calcein-AM release test. Calcein-AM was purchased from Invitrogen as a 1 mg/mL solution in dimethyl sulfoxide (DMSO; Sigma, St. Louis, MO, USA). MDA-231 and MDA-231/NF cells were grown in 75 cm^2^ flasks and incubated for 7 hr at 37°C in HBSS only or in HBSS containing 14.8 MBq of I-131. After 7 hr, I-131 containing medium was removed and cells were washed twice with HBSS. Cells were grown for three days. Then, irradiated or non-irradiated MDA-231/NF cells were re-suspended in the complete medium at a final concentration of 10^6^ cells/mL and were incubated with 5 *µ*M Calcein-AM for 20 min at 37°C, allowing Calcein-AM to enter the target cells. The labeled target cells were then washed twice and dispensed at a concentration of 1×10^4^ target cells*/*well in a round-bottom, 96-well plate (Nunc, Roskilde, Denmark). The effectors were distributed in triplicate at effector : target (E : T) cell ratios from 2.5 : 1, 5 : 1, and 10 : 1 with at least three replicate wells for spontaneous (only target cells in complete medium) and maximum release (only target cells in medium plus 1% NP-40 cell lysis buffer). After incubation at 37°C in 5% CO_2_ for 4 hr, each supernatant was harvested and transferred into new plates. Samples were measured using a multilabelcounter (VICTOR3, Perkin Elmer, San Diego, CA, USA) (excitation filter: 485±9 nm; band-pass filter: 530±9 nm). Data were expressed as arbitrary fluorescent units. NK cell cytotoxicity was calculated using the following equation:
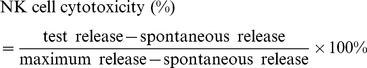



### *In vivo* Animal Experiments

Twelve mice were divided into four groups for assessment of therapeutic effects (three mice per group); the experimental groups were referred to as the control, I-131, NK, and combined groups. In 12 mice, MDA-231/NF cells (5×10^5^) were implanted subcutaneously into the right flank.

In the control group, intravenous injection of PBS was administered at 14 days post-challenge. In the I-131 group, intraperitoneal injection of 29.6 MBq of I-131 was administered at 14 days post-challenge. In the NK group, NK92-MI cells (5×10^6^) were injected intravenously via tail vein at 17 and 18 days. A total of two doses were administered to each mouse with two days apart. The combined group received treatment with both I-131 at 14 days and NK92-MI cells at 17 and 18 days.

Bioluminescence imaging was performed using the IVIS lumina II imaging system (Caliper). From 14, 24, and 34 days post-challenge, mice received intraperitoneal injection with 100 µL of D-luciferin (30 mg/mL). After 5 min, mice were placed individually in the specimen chamber and images were then acquired. Grayscale photographic images and bioluminescent color images were superimposed using LIVING IMAGE, version 2.12 (Caliper, Alameda, CA, USA), and IGOR image analysis FX software (WaveMetrics, Lake Oswego, OR, USA). Bioluminescent signals were expressed in units of photons per cm^2^ per second per steradian (P/cm^2^/sec/sr).

### Statistical Analysis

All data are expressed as means ± SDs and are representative of at least two separate experiments. The unpaired Student’s t test and ANOVA analysis were used for determination of statistical significance. P values of <0.05 were considered statistically significant.

## Results

### Verification of MDA-231 Expressing hNIS and Effluc Genes

**Figure 1:**
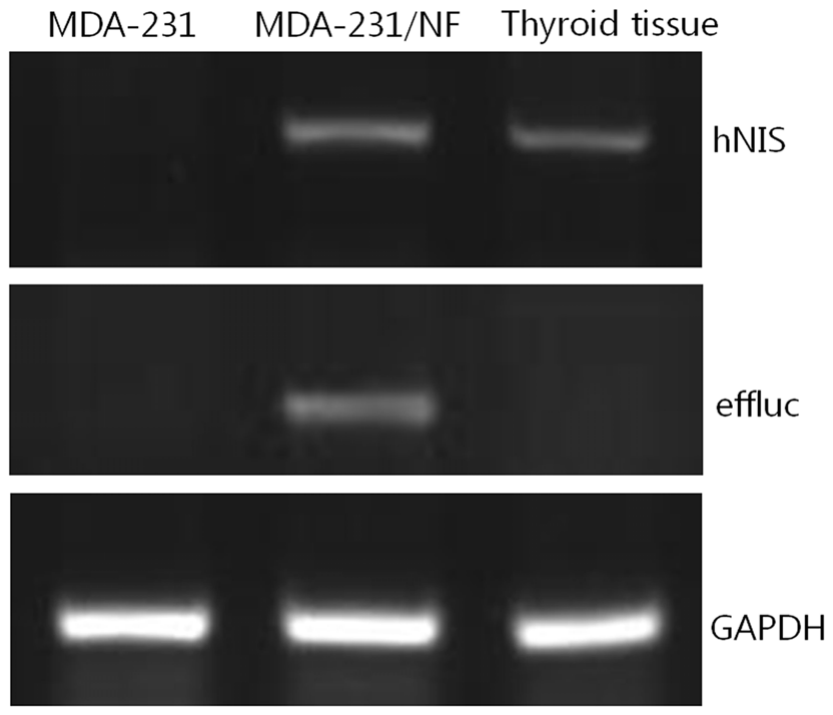
RT-PCR analysis of human sodium/iodide symporter (hNIS) and enhanced firefly luciferase (effluc) gene expression in MDA-231, MDA-231/NF cells and human thyroid tissue. RT-PCR revealed hNIS mRNA expression in MDA-231/NF cells and human thyroid tissue, and effluc mRNA expression in MDA-231/NF cells. RT-PCR fragments have lengths of 583 bp and 316 bp for hNIS and effluc in MDA-231/NF cells; however, these bands do not appear in MDA-231 cells.

Expression of hNIS and effluc genes of MDA-231/NF cells was confirmed by RT-PCR analysis. Human thyroid tissue was used as positive control for hNIS expression in MDA-231/NF cells. RT-PCR revealed hNIS mRNA expression in MDA-231/NF and human thyroid tissue. RT-PCR fragments had lengths of 583 bp and 316 bp for hNIS and effluc in MDA-231/NF cells, however, these bands did not appear in MDA-231 cells (Figure 1).

**Figure 2:**
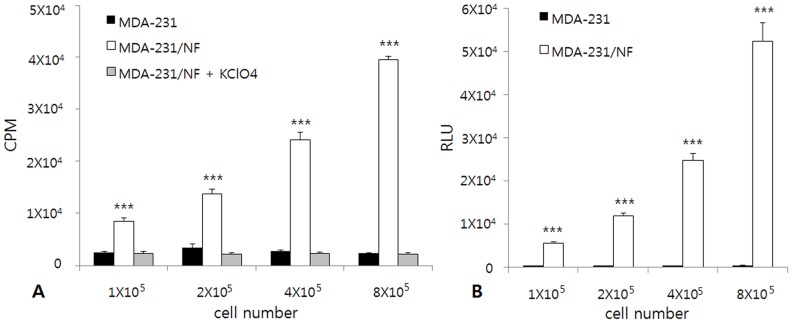
*In vitro* I-125 uptake assay and luciferase assay in MDA-231 and MDA-231/NF cells. (A) I-125 uptake by MDA-231/NF cells increased according to cell number. I-125 uptake by MDA-231 cells remained at the basal level. *** p<0.001 compared with MDA-231 and MDA-231/NF cells blocked by KClO_4._ (B) Bioluminescence signals of MDA-231/NF cells increased according to cell number. Bioluminescence signal of MDA-231 cells remained at the basal level. CPM: count per minute, RLU: Relative Light Units, *** p<0.001 compared with MDA-231 cells.

*In vitro* I-125 uptake assay showed that I-125 uptake by MDA-231/NF cells increased according to cell number, whereas I-125 uptake by MDA-231 cells and MDA-231/NF cells blocked by KClO_4_ remained at the basal level (Figure 2A). I-125 uptake in MDA-231/NF cells was 17-fold higher than the uptake observed in MDA-231 cells. The presence of 1mM KClO_4_ inhibited I-125 uptake completely in MDA-231/NF cells. *In vitro* luciferase assay was performed for MDA-231/NF and MDA-231 cells. Bioluminescence signals of MDA-231/NF cells increased according to cell number, whereas bioluminescence signals of MDA-231 cells remained at background level (Figure 2B). The signal intensity was approximately 1,180-fold higher in MDA-231/NF cells than in MDA-231 cells.

**Figure 3:**
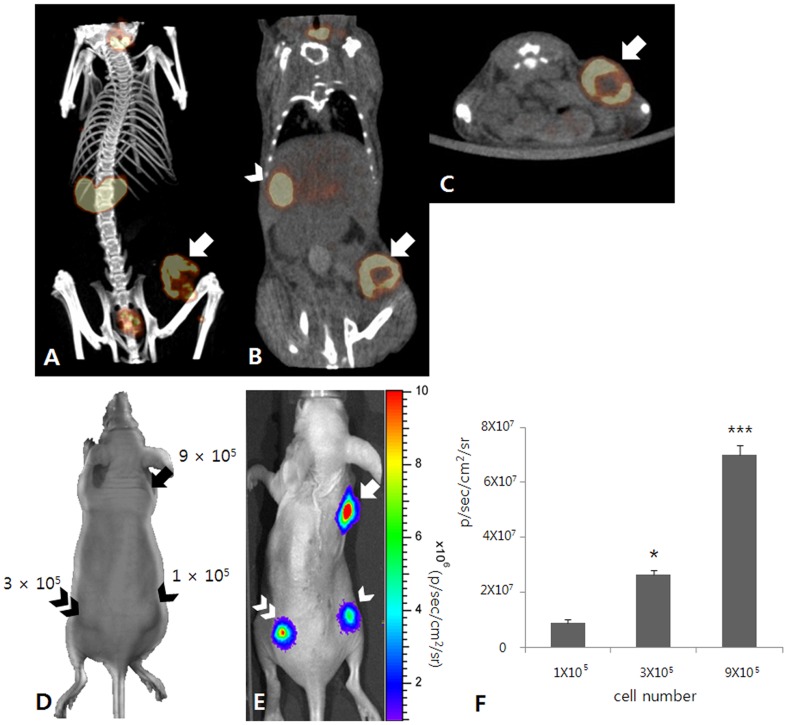
Characterization image of MDA-231/NF cells. (A–C) Tc-99m pertechnetate SPECT/CT images show focal tracer uptake (arrow) in the right flank of the MDA-231/NF tumor xenograft. There is also noted tracer uptake at the stomach (arrow head). (D–F) *In vivo* bioluminescence imaging for a mouse with MDA-231/NF cell implantation. (D) MDA-231/NF cells were implanted subcutaneously into the right hind-flank (1×10^5^; arrow head), left hind-flank (3×10^5^; double arrow head), and right fore-flank (9×10^5^; arrow) of the mouse. (E) Bioluminescence signals from implanted MDA-231/NF cells are clearly visualized in the right hind-flank (arrow head), left hind-flank (double arrow head), and right fore-flank (arrow). (F) The signal intensity from tumor cells increased with increasing cell number. * p<0.05 compared with 1×10^5^ cells, *** p<0.001 compared with 1×10^5^ and 3×10^5^ cells.

To evaluate the functional expression of the hNIS gene in a tumor xenograft, Tc-99m pertechnetate SPECT/CT scan was performed in a mouse animal model. Focal tracer uptake was observed in the right flank of the MDA-231/NF tumor xenograft (Figure 3). To evaluate the functional expression of the effluc gene in the tumor xenograft, bioluminescence imaging was performed in a mouse animal model. Bioluminescence signals from implanted MDA-231/NF cells were clearly visualized in the right fore-flank and bilateral hind-flanks. The signal intensity from tumor cells increased with an increasing number of implanted cells.

### Phenotypic Modulation of Cancer Cells by I-131 Therapy *in vitro*

**Figure 4:**
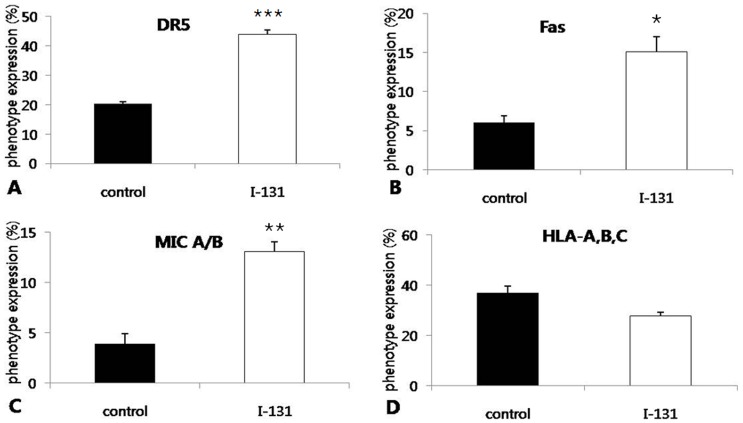
Phenotype analysis in MDA-231/NF cells by flow cytometry. (A, B and C) The levels of DR5, Fas, and MIC A/B expression in tumor cells that received I-131 were significantly higher than in tumor cells received. (D) I-131 therapy resulted in decreased expression of HLA-A,B,C, but without statistical significance. * p<0.05, ** p<0.005, *** p<0.001 compared with control.

To investigate the effect of I-131 therapy at surface levels of Fas, DR5, MIC A/B, and HLA-ABC of MDA-231/NF cells *in vitro*, flow cytometric analysis was performed in cells that received I-131 or PBS. The levels of Fas and DR5 were significantly higher in irradiated cells than in non-irradiated cells, respectively (15.1% vs. 6.0% and 44.0% vs. 20.3%; p = 0.027 and p<0.001). In addition, the level of MIC A/B was significantly higher in irradiated cells than in non-irradiated cells (13.1% vs. 3.9%; p = 0.004) (Figure 4). Although statistically not significant, the level of HLA-ABC was lower in irradiated cells than in non-irradiated cells (27.8% vs. 37.0%; p = 0.061).

### Enhancement of NK Cell Cytotoxicity by I-131 Therapy *in vitro*

**Figure 5:**
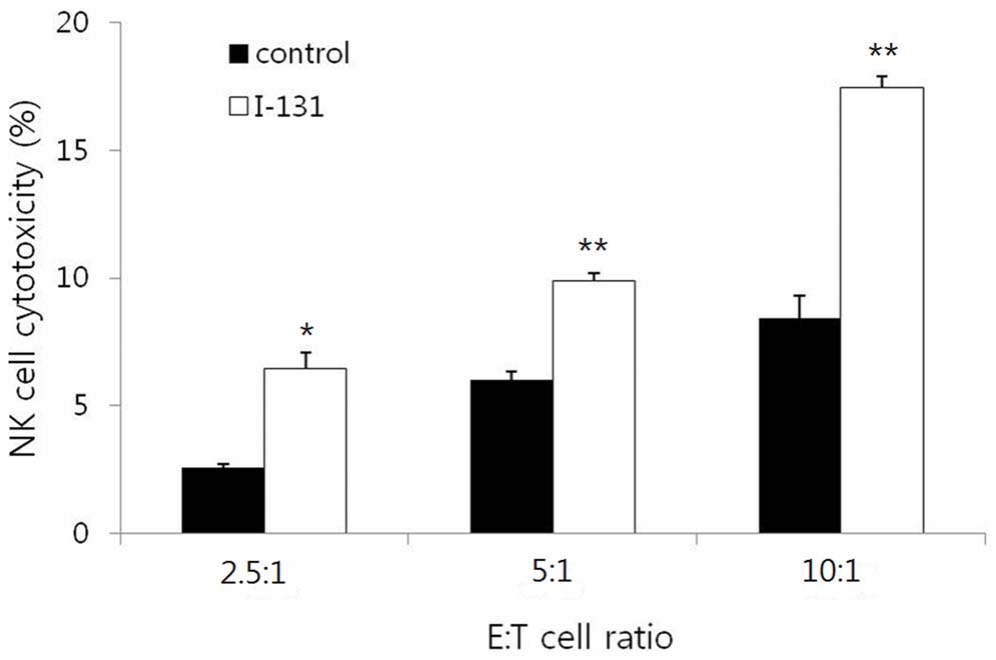
*In vitro* Cytotoxicity assay using Calcein-AM. NK cell cytotoxocity is significantly increased according to effector : target cell (E : T) ratio in the control and I-131 groups. NK cell cytotoxicities were significantly higher in irradiated cells than in non-irradiated cells at all E : T ratios of 2.5 : 1, 5 : 1, and 10 : 1. * p<0.05, ** p<0.005 compared with control.

To determine whether I-131 therapy can enhance NK cell cytotoxicity to breast cancer cells, the cytotoxicity assay was performed in MDA-231/NF cells that received I-131 or PBS at E : T cell ratios of 2.5 : 1, 5 : 1, and 10 : 1. Cytotoxicity to the target cells was significantly increased by high E : T ratio in both non-irradiated and irradiated MDA-231/NF cells (p<0.001 and p = 0.001). NK cell cytotoxicities to non-irradiated cells were 2.6%, 6.0%, and 8.4% at an E : T ratio of 2.5 : 1, 5 : 1, and 10 : 1. NK cell cytotoxicities to the irradiated cells were 6.4%, 9.9%, and 17.5% at an E : T ratio of 2.5 : 1, 5 : 1, and 10 : 1 respectively (Figure 5). NK cell cytotoxicities were significantly higher in irradiated cells than in non-irradiated cells at all E : T ratios of 2.5 : 1, 5 : 1, and 10 : 1, respectively (p = 0.020, p = 0.001 and p = 0.003).

### Enhancement of NK Cell Cytotoxicity by I-131 Therapy *in vivo*

The mice were divided into four groups (control, I-131, NK, and combined groups) and PBS, I-131, NK92-MI or I-131 and NK92-MI were applied to each group. Tumor burden was monitored by bioluminescence imaging 14, 24, and 34 days after tumor inoculation.

**Figure 6:**
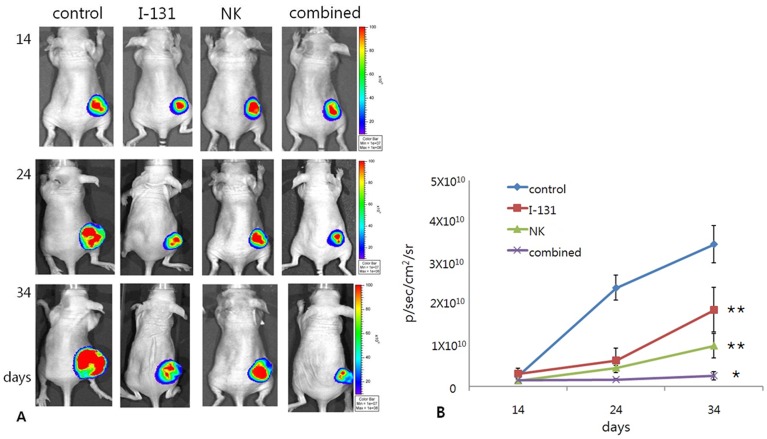
Tumor burdens monitored by bioluminescence imaging 14, 24, and 34 days after tumor inoculation. (A and B) The tumor signals in the I-131 and NK groups were significantly lower than those in the control group at 34 days. The combined group showed the lowest stationary tumor signal over time, which was significantly lower than that in the I-131 and NK groups at 34 days. * p<0.05 compared with I-131 and NK groups,** p<0.005 compared with control group.

Mice in the control group who received PBS showed a continuous increase in their tumor signal. The I-131 and NK groups showed moderate increases in tumor signals and the tumor signals of the I-131 and NK groups were consistently lower than that of the control group and significantly lower than that of the control group at 34 days, respectively (p = 0.017 and p = 0.001). The combined group showed a stationary tumor signal over time, which was consistently lower than that of the I-131 and NK groups, and significantly lower than that of the I-131 and NK groups at 34 days, respectively (p = 0.030 and p = 0.038) (Figure 6). Tumor burdens measured by bioluminescence imaging did not differ significantly between the NK and I-131 groups (p = 0.155).

## Discussion

Contemporary management of breast cancer with early detection, newer local control techniques, improved chemotherapy regimens, and targeted treatments has resulted in immense gains in survival in individuals with breast cancer [21]. Unfortunately, the triple negative breast cancers, a subset of breast cancers clinically defined by the absence of the estrogen receptor, progesterone receptor, and HER-2 over expression, have a higher propensity to metastasize to distant visceral organs, and have a worse outcome with a high rate of recurrence after adjuvant treatment [4]. Thus, the need for development of successful therapeutic options in an attempt to improve the outcome is urgent. An attractive approach to reducing the rate of recurrences in these individuals is use of immunotherapeutic strategies [6], [22]. However, because of the complexities of tumor microenvironments, effective therapeutic effects may not be feasibly achieved in immunotherapy. Several researchers have reported obstacles to successful immunotherapy, such as tumor-derived suppression cytokines, the absence of danger signals, loss of MHC class molecules, and low antigen levels [22], [23]. To overcome these impediments, modulation of the tumor microenvironment is essential. The current study showed that I-131 therapy can modulate microenvironment of breast cancer and improve the therapeutic effect through enhancement of NK cell cytotoxicity to tumor cells.

NK cells comprise 10–15% of all circulating lymphocytes and are also found in peripheral tissues, including the liver, peritoneal cavity, and placenta. Resting NK cells circulate in the blood; however, following activation by cytokines, they are capable of extravasation and infiltration into most tissues that contain pathogen infected or malignant cells [24]. NK92 is a human NK cell line first established in 1994 from a 50-year-old male patient with an aggressive NK cell lymphoma [25]. The NK92 cell line has been examined clinically as a treatment for advanced sarcoma and leukemia [25], [26]. The parental NK92 cell line is highly dependent on the cytokine IL-2 and therapies involving these cells *in vivo* require superphysiological amounts of IL-2. However, an IL-2 independent cell line, NK92-MI, which has been shown to be virtually identical to the parental cell line, may be a more appropriate choice for clinical therapies [26], [27]. Nagashima et al. [28] reported that NK92-MI sustained proliferation in the absence of exogenously supplied IL-2 and showed greater *in vivo* anti-tumor activity in mice. The current study also showed that NK92-MI have anti-tumor activity to a breast cancer cell line *in vitro* and *in vivo* without IL-2 supplementation.

The cytotoxicity of NK cells are carried out by two main mechanisms. The first mechanism is granule-dependent cytotoxicity, where upon triggering by activating receptors [6]. Upon recognition of the ligands on the surface of the target cell surface by activating NK cell receptors, various intracellular signaling pathways drive NK cells toward cytotoxic action, which results in cytolysis of target cells [29]. When NK cells are activated by MIC A/B, which are ligands for the activating receptor NKG2D on the tumor surface, perforin and granzyme B are released to the tumor cell, resulting in mediation of apoptosis [30]. However, these processes are tightly controlled by a group of inhibitory receptors. These receptors act as negative regulators of NK cytotoxicity and inhibit the action of NK cells against ‘self’ targets. A main group of this type of receptors is NK cell immunoglobulin-like receptors (KIRs), which are mainly specific for self MHC Class-I molecules. Members of the KIR family recognize HLA-A, B and C alleles [31]. The second mechanism is the triggering of apoptosis pathways in the target cell via stimulation of death receptors by TRAIL or Fas ligand expressed on the surface of NK cells as well as secretion of TNF-α. NK cells express Fas ligand and TRAIL, which are both members of the TNF family and have been shown to induce target cell apoptosis when they bind their receptors on target cells [32]. In the current study, the levels of surface expression of Fas, DR5, and MIC A/B showed an increase on cells treated with I-131 in *in vitro* study. Up-regulation of Fas, DR5, and MIC A/B on the surface of irradiated tumor cells could explain the enhancement of NK cell cytotoxicity to tumor cells in both *in vitro* and *in vivo* studies.

Irradiation can alter immunogenecity of the tumor as well as the circumstances of the immunologic condition. Several groups have reported that radiation therapy concomitantly up-regulates the levels of Fas, DR5, and MIC A/B in several tumor cells. Ishikawa et al. [13] reported that external radiation therapy enhanced Fas and DR5 expression in glioma cell lines and cytotoxicity of NK cells was enhanced after radiation therapy. Zhou et al. [17] reported that DR5 expression was enhanced in melanoma cell lines by external radiation therapy and treatment with TRAIL resulted in significantly increased tumor cell apoptosis caused by radiation therapy. Xu et al. [33] also reported that radiation therapy up-regulated the level of MIC A/B and increased the sensitivity of NK cell killing in a pancreatic cancer cell line. However, external radiation therapy is limited in treatment of multiple metastatic lesions. Like external radiation therapy, I-131 therapy can also alter immunogenicity of tumor cells by providing radiation to the cells. In addition, I-131 therapy is applicable to treatment of multiple metastatic breast cancer, which can take up I-131 by hNIS expression [34]. Jeon et al. [15] reported that I-131 therapy can lead to up-regulated expression of Fas and enhance the killing activities of cytotoxic T cells in a colon cancer cell line. In accordance with previous studies, the present study demonstrated that I-131 therapy up-regulated the level of Fas, DR5 and MIC A/B expression in breast cancer cell line. Because the majority of breast cancers are known to express hNIS and take up iodide, it would be applicable in the clinical setting [19].

A limitation of the current study is the use of MDA-231/NF cells expressing hNIS, instead of parental MDA-231 cells, for I-131 therapy, and it is not straightforward in clinical practice. Expression of hNIS is known to be enhanced by certain agents such as a retinoic acid, and enhancement by hNIS inducible agents has been well investigated in breast cancer cells [35]–[37]. hNIS expression in breast cancer cells by the inducible agents (not by transduction of the hNIS gene) would be more appropriate in the clinical field. For clinical translation of this strategy, conduct of further studies on enhancement of NK cell cytotoxicity to parental breast cancer cells with hNIS inducible agents followed by I-131 therapy is needed.

### Conclusion

I-131 therapy would lead to up-regulation of the level of death receptors in breast cancer cells and improve therapeutic efficiency of NK cell therapy through enhancement of the cytotoxic effect of NK cells to the cancer. Enhancement of NK cell cytotoxicity by I-131 pretreatment can be applied to breast cancer patients with iodine avid metastatic lesions that are non-responsive to conventional treatments.
